# Paramedian contralateral supracerebellar infratentorial approach to thalamic cavernous malformation

**DOI:** 10.3171/2019.7.FocusVid.19167

**Published:** 2019-07-01

**Authors:** André Bortolon Bissoli, Aderaldo Costa Alves Junior, Pedro Tadao Hamamoto Filho, Marco Antonio Zanini

**Affiliations:** 1 Neurosurgery Division, Botucatu Medical School, State University of São Paulo; and; 2 Botucatu Medical School, State University of São Paulo, Botucatu, São Paulo, Brazil

**Keywords:** thalamic cavernous malformation, supracerebellar infratentorial approach, microsurgical resection, video

## Abstract

Cavernous malformations (CVMs) located in the thalamus are uncommon. However, they pose difficulties for resection because of their close proximity to eloquent areas of the brain and vascular structures, and all surgical corridors to access them are narrow. In this video, we report the case of a 19-year-old woman who presented with a long-standing history of right hemiparesis with recent deterioration. MRI revealed a large CVM located in the left thalamus, with signs of recent hemorrhage extending to the left cerebral peduncle. Resection was achieved with a paramedian contralateral supracerebellar infratentorial approach in a semisitting position, with an uneventful postoperative course.

The video can be found here: https://youtu.be/Arvu52FkHOE.

## Transcript

00:20 This video illustrates the microsurgical techniques for resection of a large-sized cavernous malformation located in the left thalamus.

00:30 The patient was a 19-year-old female which presented with sudden high-intensity headache and worsening of long-standing spastic hemiparesis of unknown etiology 6 days before admission, going from grade 4 to grade 1 motor strength.

00:49 Visual impairment was also noted, with blurring of the right visual fields.

00:56 Her CT scan has shown a large lesion in the left thalamus with hemorrhage extending inferiorly to the left cerebral peduncle, with calcifications throughout its interior.

01:10 Her MRI study later revealed a large cavernous malformation in the left thalamus with inferior extension to the mesencephalon, in the left cerebral peduncle.

01:23 Tractography is a valuable tool for identification of the corticospinal tract and choice of surgical approach; due to the proximity of the thalamus and the posterior limb of the internal capsule, most thalamic cavernous malformations displace this tract anteriorly. However, DTI imaging was not available at that time.

01:45 The semisitting position was chosen to allow a paramedian contralateral supracerebellar infratentorial approach, giving access to the cisternal portion of the left pulvinar. The operative site was marked with the aid of neuronavigation. Typical prepping and draping was performed.

02:09 A posterior linear incision was made, and muscle dissection was performed through the midline in a relatively avascular plane. The suboccipital region and the posterior arc of C1 were exposed.

02:26 A median suboccipital craniotomy with paramedial extension was performed, and venous sinus hemostasis was achieved with oxidized cellulose and fibrin glue.

02:59 A C-shaped infratentorial durotomy followed, exposing both cerebellar hemispheres.

01:13 Careful dissection of the pineal region was performed.

03:47 With further dissection we reached the cisternal portion of the left pulvinar, where hemosiderin deposits were identified.

03:57 The quadrigeminal cistern was identified, as well as the pineal gland, the vein of Galen, the left basal vein of Rosenthal, and the precentral cerebellar vein.

04:17 The operative corridor was checked with the aid of neuronavigation, aimed at the cisternal portion of the left pulvinar.

04:27 The lesion was exposed after white matter dissection. It had a typical cavernous malformation aspect.

05:00 The lesion was gradually devascularized.

05:21 We removed the cavernous malformation in a piecemeal fashion, using meticulous dissection and coagulation and avoiding excessive manipulation of the adjacent structures.

06:14 After complete removal of the lesion, careful and thorough hemostasis was performed.

06:20 It is generally reported that the PCCV can be sacrificed without any clinical symptoms, as it was necessary at the end of this case. In this final picture, it is possible to visualize the surgical corridor after dissection and removal of the cavernous malformation.

06:42 The dura was closed in a watertight fashion, followed by sutures of layers of muscle, subcutaneous tissue, and skin.

06:52 The patient had an excellent postoperative recovery.

06:58 She underwent postoperative MRI 3 months later, with good results after surgery.

07:06 Her motor strength returned to the baseline immediately after surgery, and showed even further improvement after 3 months, reaching grade 4+.

## References

[1] Kodera T Bozinov O Sürücü O Ulrich NH Burkhardt JK Bertalanffy H Neurosurgical venous considerations for tumors of the pineal region resected using the infratentorial supracerebellar approach J Clin Neurosci 18 1481 1485 2011 21917460 10.1016/j.jocn.2011.02.035

[2] Komiyama M Functional venous anatomy of the brain for neurosurgeons Jpn J Neurosurg 26 488 495 2017

[3] Mascitelli J Burkhardt JK Gandhi S Lawton MT Contralateral supracerebellar-infratentorial approach for resection of thalamic cavernous malformations Oper Neurosurg 15 404 411 2018 10.1093/ons/opy004 29490051

[4] Pandey P Westbroek EM Gooderham PA Steinberg GK Cavernous malformation of brainstem, thalamus, and basal ganglia Neurosurgery 72 573 589 2012 10.1227/NEU.0b013e318283c9c2 23262564

[5] Sindou MP Auque J Jouanneau E Neurosurgery and the intracranial venous system Acta Neurochir Suppl 94 167 175 2005 16060259 10.1007/3-211-27911-3_27

